# Nonintrusive Finger-Vein Recognition System Using NIR Image Sensor and Accuracy Analyses According to Various Factors

**DOI:** 10.3390/s150716866

**Published:** 2015-07-13

**Authors:** Tuyen Danh Pham, Young Ho Park, Dat Tien Nguyen, Seung Yong Kwon, Kang Ryoung Park

**Affiliations:** Division of Electronics and Electrical Engineering, Dongguk University, 26 Pil-dong 3-ga, Jung-gu, Seoul 100-715, Korea; E-Mails: phamdanhtuyen@gmail.com (T.D.P.); fdsarew@dongguk.edu (Y.H.P.); nguyentiendat@dongguk.edu (D.T.N.); sbaru07@dgu.edu (S.Y.K.)

**Keywords:** nonintrusive finger-vein capturing device using NIR image sensor, misalignment of finger-vein image, multiple images for enrollment, score-level fusion

## Abstract

Biometrics is a technology that enables an individual person to be identified based on human physiological and behavioral characteristics. Among biometrics technologies, face recognition has been widely used because of its advantages in terms of convenience and non-contact operation. However, its performance is affected by factors such as variation in the illumination, facial expression, and head pose. Therefore, fingerprint and iris recognitions are preferred alternatives. However, the performance of the former can be adversely affected by the skin condition, including scarring and dryness. In addition, the latter has the disadvantages of high cost, large system size, and inconvenience to the user, who has to align their eyes with the iris camera. In an attempt to overcome these problems, finger-vein recognition has been vigorously researched, but an analysis of its accuracies according to various factors has not received much attention. Therefore, we propose a nonintrusive finger-vein recognition system using a near infrared (NIR) image sensor and analyze its accuracies considering various factors. The experimental results obtained with three databases showed that our system can be operated in real applications with high accuracy; and the dissimilarity of the finger-veins of different people is larger than that of the finger types and hands.

## 1. Introduction

Recent developments have led to the widespread use of biometric technologies, such as face, fingerprint, vein, iris, and voice recognition, in a variety of applications in access control, financial transactions on mobile devices, and automatic teller machines (ATMs) [1,2,3,4]. Among them, finger-vein recognition has been highlighted because it can overcome several drawbacks of other biometric technologies, such as the effect of sweat, skin distortions, and scars in fingerprint recognition, or the effect of poses and illumination changes in face recognition. Moreover, a finger-vein recognition system is cost effective in comparison, and offers high accuracy together with the advantages of fake detection and a bio-cryptography system [5]. Finger-vein recognition uses the vascular patterns inside human fingers to uniquely identify individuals. Vein imaging technology relies on the use of near infrared (NIR) illuminators at a wavelength longer than about 750 nm, because the deoxyhemoglobin in veins absorbs light in this range [6,7]. Previous work on finger-vein recognition include research aimed at enhancing vein image quality, increasing recognition accuracy by various feature extraction methods, considering finger veins as a factor for individual recognition in multimodal systems, as well as detecting fake finger veins. The research on finger-vein image enhancement, which is based on a software algorithm, can be classified into restoration-based and non-restoration-based methods [7,8]. The restoration-based methods proposed by Yang *et al.* [9,10,11] were able to produce enhanced finger-vein images by considering the effect of the layered structure of skin and restored the images by using a point-spread function (PSF) model [10], and a biological optical model (BOM) [11]. In the non-restoration-based approaches, Gabor filtering was popularly used [6,7,8,12,13]. Yang *et al.* introduced an enhancement method that uses multi-channel even-symmetric Gabor filters with four directions to strengthen the vein information in different orientations [6]. A study by Park *et al.* [8] led to the proposal of an image enhancement method using an optimal Gabor filter based on the directions and thickness of the vein line. An adaptive version of the Gabor filter was used in the research of Cho *et al.* [12] to enhance the distinctiveness of the finger-vein region in the original image. The Gabor filter was also used in combination with a Retinex filter, by using fuzzy rules in the method proposed by Shin *et al.* [7]. Zhang *et al.* proposed gray-level grouping (GLG) for the enhancement of image contrast, and a circular Gabor filter (CGF) for the enhancement of finger-vein images [13].

Pi *et al.* proposed a quality improvement method based on edge-preserving and elliptical high-pass filters capable of maintaining the edges and removing blur [14]. In addition, Yu *et al.* proposed a fuzzy-based multi-threshold algorithm considering the characteristics of the vein patterns and skin region [15].

Work has also been conducted on extracting and combining various features from finger-vein images to increase the quality of the recognition results [16,17,18,19]. In [16], they used both the global feature of the moment-invariants method and Gabor filter-based local features. In the method proposed by Lu *et al.* [17], eight-channel Gabor features were extracted and analyzed prior to application to score-level fusion to obtain the final matching score. Qian *et al.* [18] proposed a finger-vein recognition algorithm based on the fusion of score level moment invariants by the weighted-average method. In [19], Yang *et al.* proposed a binary feature for finger-vein matching, termed personalized best bit map (PBBM), which was extracted based on the consistent bits in local binary pattern (LBP) codes. Finger-vein recognition was also considered as a sub-system in multimodal biometric systems [20,21,22,23] along with other individual recognition methods to compensate for the drawbacks of each of the recognition methods. The results of finger-vein and fingerprint recognitions were matched and combined by using various methods, such as decision level fusion of “AND” or “OR” rules as in [20], a support vector machine (SVM) as in [21], or score level fusion as in [22]. He *et al.* [23] proposed a multimodal biometric system by considering the three biometric characteristics of fingerprint, face, and finger-vein, and evaluated the performance of the system with the use of sum rule-based and SVM-based score level fusion. The research on finger-vein recognition has also taken counterfeit vein information into account, as in [24,25]. In the anti-spoofing system for vein identification in [24], live fingers were detected by continuously capturing successive heart-rate-based images and then examining the details in the series of images. Nguyen *et al.* [25] proposed an image-analysis method for fake finger-vein detection based on Fourier transform and wavelet transforms.

A number of research efforts on finger-vein recognition have considered the quality of the preprocessed images, as well as the effectiveness of the matching features. However, the evaluation of the discriminant factors on finger-vein information, such as the differences between people, left and right hands, and the type of finger, has not received much attention. In our research, we propose a nonintrusive finger-vein capturing device.

**Table 1:** Comparison of the proposed method with previous methods.

Category	Methods	Strengths	Weaknesses
Accuracy evaluation without considering the various factors of people, hands, finger types, and the number of images	EER or ROC curve-based evaluation of finger-vein recognition with the assumption that the veins from different hands or finger types are different classes without comparing the dissimilarity of finger-vein among people, hands, and finger types [7,8,9,11,16,17,18,19,20,21,22,23,25]	New methods for enhancing finger-vein images with feature extraction or score fusions for enhancing the recognition accuracy are proposed	Assuming the veins from different hands or finger types are different classes without any theoretical or experimental ground
Accuracy evaluation according to people, hands, finger types, and the number of images	The dissimilarity of finger-veins among people, hands, and finger types are quantitatively evaluated (Proposed method)	Providing the experimental ground for the dissimilarity of finger-veins among people, hands, and finger types	Not providing the experimental ground for the dissimilarity of palm-veins or hand dorsal veins among people and hands

Our research is novel in the following three ways compared to previous work.

∙ We propose a nonintrusive finger-vein capturing device using a small-sized web-camera and NIR light-emitting diodes. To reduce the misalignment of captured images while ensuring minimal user inconvenience, two guiding bars for positioning the fingertip and side of the finger were attached to the device.
∙ The accuracies of recognition were compared by assuming that images from the same person, the same hand, and the same finger types form the same classes. Based on the receiver operational characteristic curve, equal error rate, authentic and imposter matching distributions, and d-prime value, the dissimilarity of finger-veins among people, hands, and finger types are quantitatively evaluated.
∙ The accuracies of recognition are compared according to the number of finger-vein images combined by score-level fusion for recognition, and the number of images for enrollment.

Table 1 presents a comparison of the proposed method with previous methods.

The remainder of this paper is organized as follows. Section 2, explains the details of the proposed method and Section 3, shows the experimental results and discussions. Finally, the conclusions and opportunities for future work are given in Section 4.

## 2. Finger-Vein Recognition and Evaluation Method

### 2.1. Overview of the Finger-Vein Recognition System

**Figure 1:**
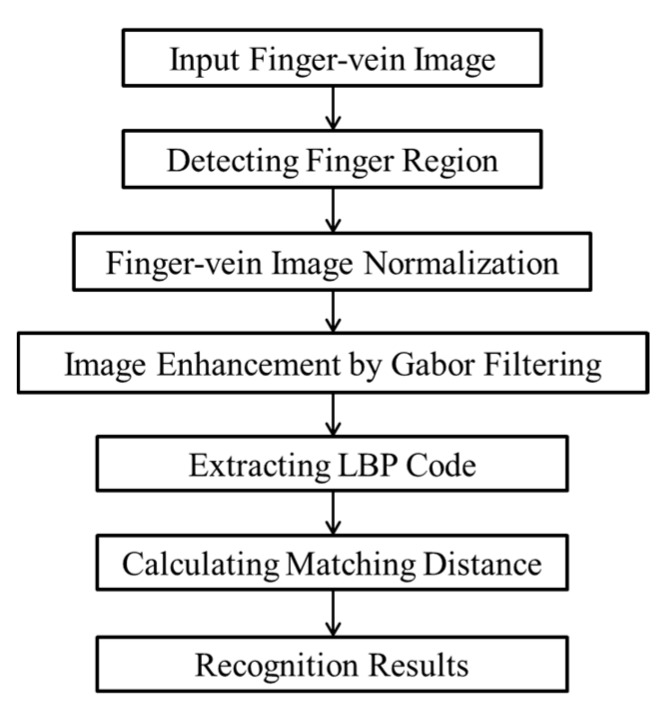
Flowchart of the experimental procedure of our research.

An overview of the proposed method is shown in Figure 1. Because the input finger-vein image consists of two parts, *i.e.*, the finger region containing the finger-vein information and the background region, the method to detect the finger region is first applied in order to remove the background, which contains unnecessary information. In the next step, based on the detected upper and lower finger boundaries detected in the previous step, the segmented finger region is stretched into a rectangular form in the normalization step. The processing time is reduced by obtaining a sub-sample of the stretched finger-vein image to reduce the size of the image. Before the recognition features are extracted, the quality of the finger-vein image is enhanced by using Gabor filtering, subsequent to which the preprocessed image is applied to the feature extraction step using the local binary pattern (LBP) method. In the next step, the hamming distance (HD) is calculated to determine the matching distance between the extracted code features of the input finger-vein image and the enrolled image. The input finger-vein image is then classified as either being genuine or being that of an imposter by using the enrolled data based on the matching distance.

### 2.2. Finger Region Detection and Normalization

**Figure 2:**
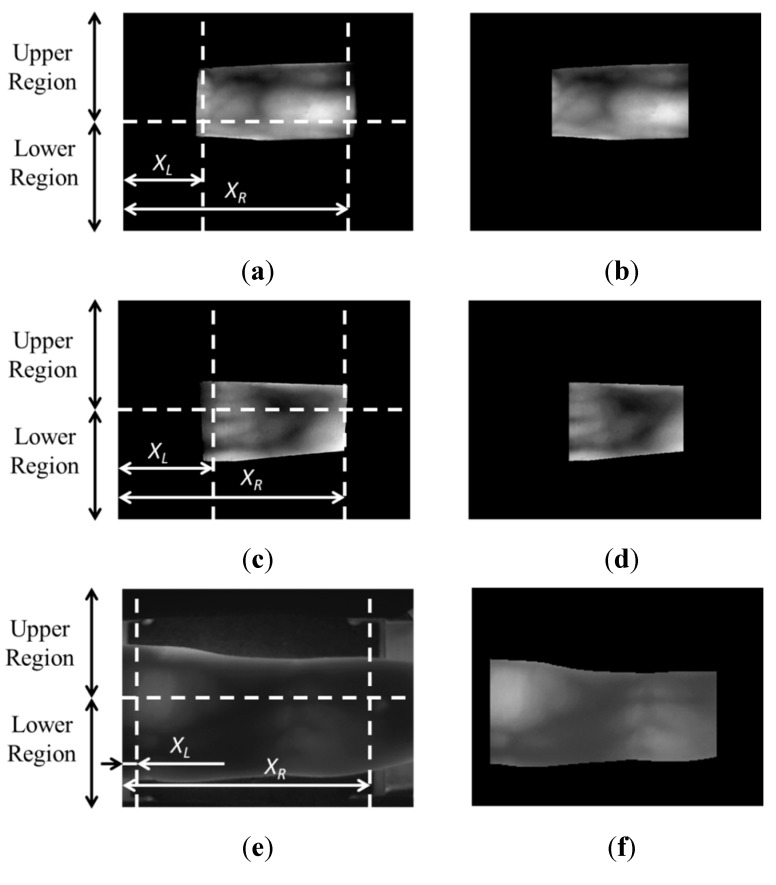
Examples of input finger-vein images and finger-region detection results obtained with images from the three databases: Original images from the (**a**) good-quality; (**c**) mid-quality; and (**e**) open databases with their corresponding finger-region detection results shown in (**b**,**d**,**f**), respectively.

As shown in Figure 2, a captured finger-vein image consists of the background surrounding the finger region, which contains the vein pattern, which is used for recognition purposes, and which has higher gray levels than the background. The background is removed from the captured image by detecting the four boundaries of the finger region consisting of the left and right boundaries in the horizontal direction, and upper and lower boundaries in the vertical direction, based on previous research [7]. In the images from the three databases, the left and right finger region boundaries are restricted by the size of the hole in the device for capturing the finger-vein image. Detailed explanations of the three databases and the device are provided in Section 3. As such, the values of *X_L_* and *X_R_*, which determine the left and right boundaries, as shown in Figure 2, are experimentally defined for the three databases. In the case of the good-quality database with 640 × 480 pixel images, the values of *X_L_* and *X_R_* are 180 and 480 pixels, respectively. For the mid-quality database with the same image size, the values of *X_L_* and *X_R_* are 220 and 470 pixels, respectively. The third (open) database, which consists of images with a size of 320 × 240 pixels, *X_L_* and *X_R_* are 20 and 268 pixels, respectively.

The 1st (Figure 2a) and 2nd database (Figure 2c) are collected by our lab-made devices (see Section 3). In our devices, each person puts his or her finger on the hole of the upper-part of device, and the size of the hole in the device for capturing the finger-vein image is fixed and limited in order to remove the effect by the environmental light into the captured image. Therefore, the part of the finger area can be acquired in the image, and the positions of left and right finger boundaries are restricted and same in all the captured images as shown in Figure 2a,c. Therefore, in order to enhance the processing speed of segmenting the finger area from the image, we use the pre-determined *X_L_* and *X_R_* values as the horizontal (X) position of the left and right boundary of the finger area, respectively.

In case of the 3rd database (Figure 2e), although the whole finger area can be acquired in the image, the left and right-most areas of finger are so dark (caused by the insufficient illumination of NIR light) that these areas are difficult to be used for finger-vein recognition. Therefore, we use the part of finger area by removing these left and right-most areas, based on pre-determined *X_L_* and *X_R_* values. The positions of the left and right boundaries can be automatically segmented with the 3rd database, but these positions can be different from each other among images, according to the performance of the segmentation algorithm of the finger area. The main goal of our research is not focused on the segmentation algorithm but on comparing the accuracies of recognition by assuming that images from the same person, the same hand, and the same finger types form the same classes. In addition, another goal is to compare the accuracies of recognition according to the number of finger-vein images combined by score-level fusion for recognition, and the number of images for enrollment. Therefore, we use the part of finger area by removing these left and right-most areas, based on pre-determined *X_L_* and *X_R_* values.

**Figure 3:**
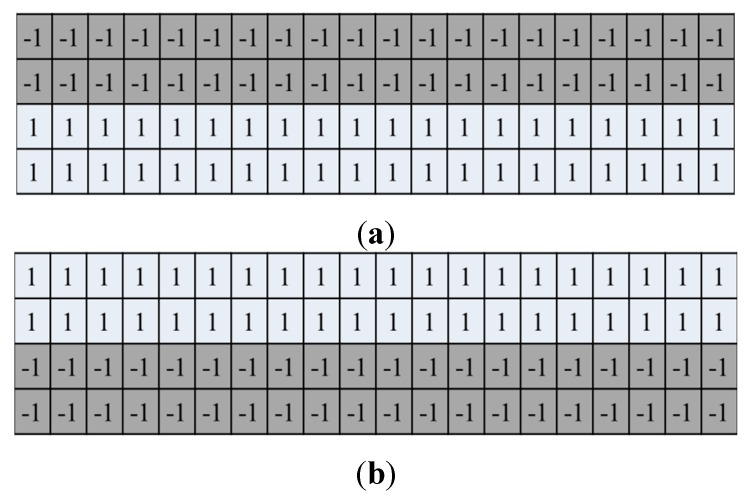
Masks for detecting finger-region boundaries in the vertical direction: Masks for detecting (**a**) the upper boundary; and (**b**) the lower boundary of the finger region.

Then, two masks of 4 × 20 pixels, which are shown in Figure 3, were used to detect the upper and lower boundaries of the finger region. Because the gray level of the background region is lower than that of the finger region, as shown in Figure 2, the value that was calculated by using the masks in Figure 3 is maximized at the position of the finger boundary. Examples of the finger region detection results are given in Figure 2b,d,f.

**Figure 4:**
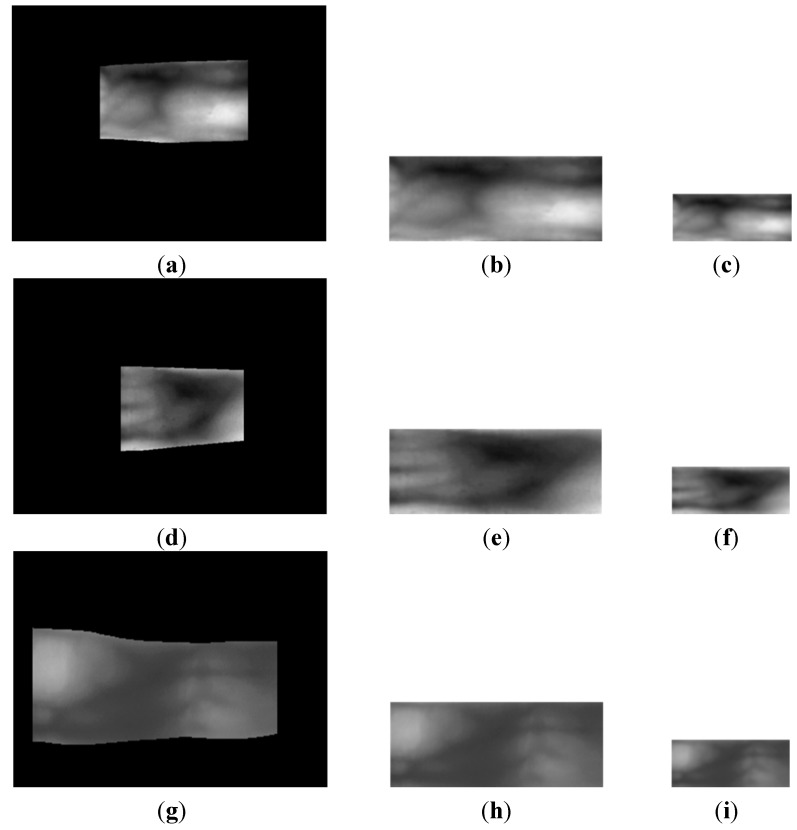
Linear stretching and sub-sampled results of finger-vein images from the three databases: Detected finger-region image from the (**a**) good-quality; (**d**) mid-quality; and (**g**) open databases, with their corresponding 150 × 60 pixel stretched images shown in (**b**,**e**,**h**), respectively, and their corresponding 50 × 20 pixel sub-sampled images shown in (**c**,**f**,**i**), respectively.

Based on the detected finger boundaries, the finger-vein image is normalized to the size of 150 × 60 pixels by using a linear stretching method, and it is then sub-sampled to produce a 50 × 20 pixel image to enhance the processing speed [7]. This is done by averaging the gray values in each 3 × 3 pixel block of the 150 × 60 pixel image. Figure 4 shows examples of the normalization results of the images in Figure 2.

### 2.3. Four-Directional Gabor Filtering

**Figure 5:**
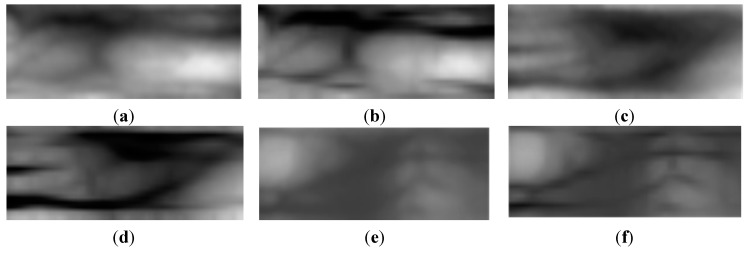
Gabor filtering results of the 50 × 20 pixel sub-sampled images from the three databases: Sub-sampled image from the (**a**) good-quality; (**c**) mid-quality; and (**e**) open databases with their respective Gabor filtered images shown in (**b**,**d**,**f**).

Gabor filtering has been popularly used in finger-vein recognition for enhancing the image quality [6,7,8]. In this research, we apply a four-directional Gabor filter to the 50 × 20 pixel sub-sampled image prior to extracting the finger-vein code to enhance the distinctiveness of the vein image. Gabor filtering of the sub-sampled image could also be helpful to reduce the processing time compared to that of the original finger-vein image [7,8]. A two-dimensional Gabor filter can be represented as follows [6,7,8]:

(1) $$G(x,y)=\frac{1}{2\pi \sigma_{x}\sigma_{y}}\text{exp}\left\{-\frac{1}{2}\left(\frac{x_{\theta }^{2}}{\sigma_{x}^{2}}+\frac{y_{\theta }^{2}}{\sigma_{y}^{2}}\right)\right\}\text{exp}\left(j2\pi f_{0}x_{\theta }\right)$$

with

$$\left[\begin{array}{c} x_{\theta }\\ y_{\theta } \end{array}\right]=\left[\begin{array}{cc} \text{cos}\theta & \text{sin}\theta \\ -\text{sin}\theta & \text{cos}\theta \end{array}\right]\left[\begin{array}{c} x\\ y \end{array}\right]$$

where $j=\sqrt{-1}$, θ is the direction, and *f*_0_ is the central frequency of the Gabor kernel. The two coordinates (*x*, *y*) are rotated to *x*_θ_ and *y*_θ_, respectively, and on each coordinate, the spatial envelopes of the Gaussian function are represented by σ_*x*_ and σ_*y*_, respectively. By eliminating the imaginary part of the Gabor filter, the real part, namely the even-symmetric Gabor filter, is used in this research because of the effectiveness with which it processes time. An even-symmetric Gabor filter is represented as Equation (2) as follows [6,7,8]:

(2) $$G_{k}^{E}(x,y)=\frac{1}{2\pi \sigma_{x}\sigma_{y}}\text{exp}\left\{-\frac{1}{2}\left(\frac{x_{\theta_{k}}^{2}}{\sigma_{x}^{2}}+\frac{y_{\theta_{k}}^{2}}{\sigma_{y}^{2}}\right)\right\}\text{cos}(2\pi f_{k}x_{\theta_{k}})$$

with

$$\theta_{k}=k\pi /4;\;k\;=\;1,\;2,\;3,\;4$$

where *k* is the index of the directional channel, and θ_*k*_ and *f_k_* represent the orientation and spatial frequency of the *k*th channel, respectively. Based on previous research [6], the optimal parameters of *f_k_*, σ_*x*_, and σ_*y*_, are determined to be 0.2, 2.38, and 2.38, respectively, for the four channels in the 0°, 45°, 90°, and 135° directions of the Gabor filter applied to the sub-sampled image of 50 × 20 pixels. A convolution operation is applied to an input finger-vein image with the Gabor filter of the four channels to obtain the filtered image in the form of four separated convolution result images. These images are then combined by selecting, at each pixel position, the pixel with the lowest gray-level value among the four pixels of the four result images to be the final result of Gabor filtering, because, generally, the vein line is darker than the skin region [7]. Figure 5 provides example results of four-directional Gabor filtering on the sub-sampled images in Figure 4c,f,i.

### 2.4. Finger-Vein Code Extraction Using LBP and Matching

**Figure 6:**
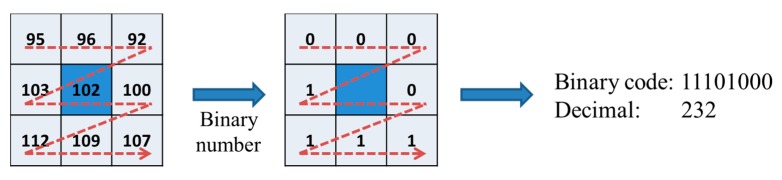
LBP operator.

The binary codes are extracted from the quality enhanced finger-vein image by using the LBP method, which was selected based on its high performance [7]. This method encodes the difference between the gray level of each central pixel (*I_C_*) and that of its neighboring pixels (*I_N_*) to the binary values of 0 or 1, as described by Equation (3) and illustrated in Figure 6. For each pixel position in a 50 × 20 pixel image, an 8-bit code string is extracted. Consequently, for each finger-vein image, a 6912-bit binary code (8 bits × 48 columns × 18 rows) is generated by the LBP operator.

(3) $$\begin{array}{l} LBP\left(x_{C},y_{C}\right)=\sum_{k=0}^{7}S\left(I_{N}-I_{C}\right)\cdot 2^{k}\\ S(t)=\{\begin{array}{lll} 1 & & if\;\;\;i\geq 0\\ 0 & & otherwise \end{array} \end{array}$$

The matching distance is calculated by using the HD between the enrolled and input LBP binary codes. In this research, we used the normalized version of the Hamming distance on all of the 6912 bits of each finger-vein image as the following Equation (4) [7]:

(4) $$HD=\frac{VCE⊕VCI}{N}$$

where *VCE* and *VCI* are the binary codes extracted from the enrolled and input images, respectively, ⊕ is the Boolean exclusive OR (XOR) operator, and *N* is the total number of bits (6912).

## 3. Experimental Results

### 3.1. Proposed Finger-Vein Capturing Device

**Figure 7:**
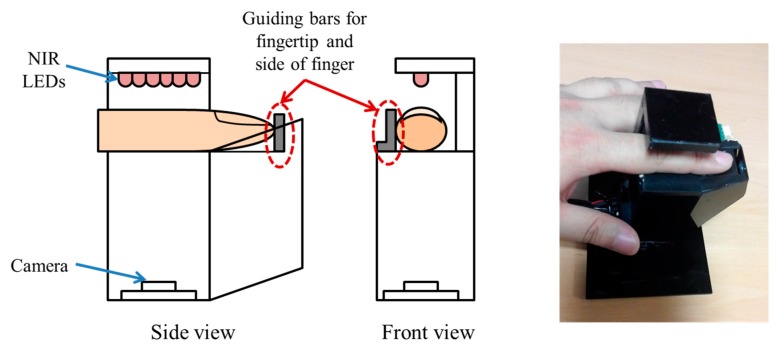
Proposed finger-vein capturing device used to build a good-quality database.

Figure 7 depicts our finger-vein capturing device. This device consists of six NIR light-emitting diodes (LEDs) operating at a wavelength of 850 nm and a webcam (Logitech Webcam C600) [26]. Alignment of the input finger-vein image in the capturing process was achieved by attaching two bars to the device to guide the positioning of the fingertip and the side of the finger. This was done to ensure a high similarity between images acquired from the same finger of an individual and thus, increase the matching accuracy. By adding the guiding bars, our device is able to acquire finger-vein images for each person non-intrusively. This enabled us to create a good-quality finger-vein database with enhanced alignment of the finger position, and to use the database for the following experiments.

### 3.2. Performance Evaluation on Three Databases

For this research, we used three different finger-vein databases to evaluate the factors that affect the matching accuracy. The first database was created by collecting finger-vein data from 20 people using the device proposed in Section 3.1. [27]. For each person, the vein images of the index, middle, and ring fingers on both the left and right hands were captured 10 times with an image resolution of 640 × 480 pixels. The total number of images in our database was 1200 (20 people × 2 hands × 3 fingers × 10 images). Because the finger alignment and image quality of the images in this database were strictly assured, it was considered a good-quality database.

**Figure 8:**
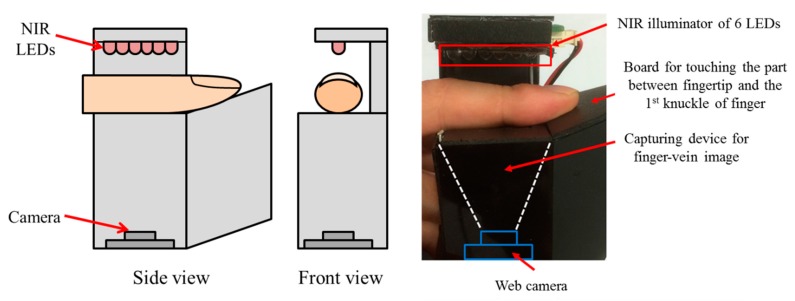
Device for capturing finger-vein images for the second (mid-quality) database.

**Figure 9:**
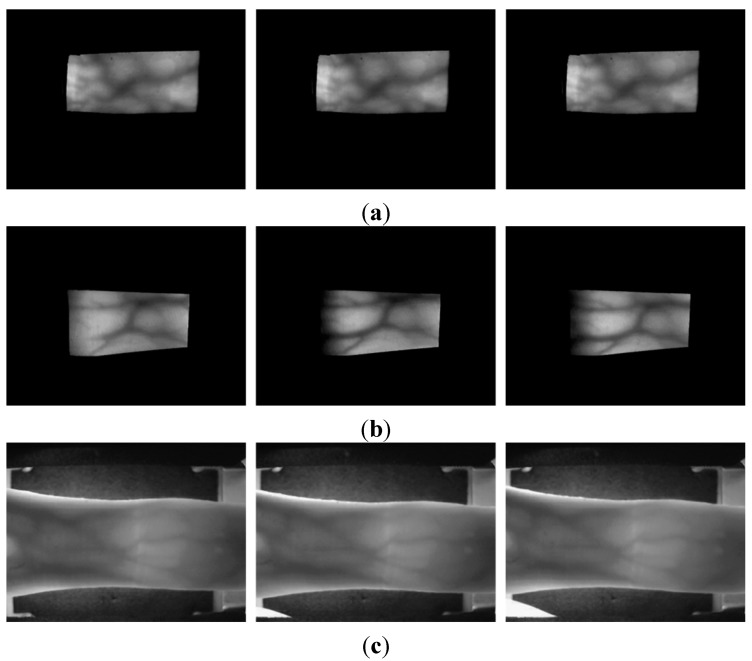
Input images of different trials from the same finger of one individual from each database: (**a**) good-quality; (**b**) mid-quality; and (**c**) open database.

In addition, we used two other databases, the first of which was constructed by selecting the vein images of six fingers among the images of 10 fingers in the database I (which were collected by the finger-vein capturing device without the guiding bar in previous research [7]). The device, which was used for collecting the database I [7], is shown in Figure 8. Because the guiding bar was absent, the misalignment among trial images of each finger in this database is relatively high; therefore, this was considered mid-quality database. In detail, each people puts his or her finger on the hole of the upper-part of device, and the size of the hole in the device for capturing the finger-vein image is fixed and limited in order to remove the effect by the environmental light into the captured image. Therefore, the part of finger area can be acquired in the image as shown in Figure 9a,b. Consequently, it is often the case that some part of the finger area (which is seen in the enrolled image) is not seen in the recognized image, which cannot be compensated by preprocessing step and can reduce the accuracy of recognition. In order to solve this problem, we propose a new device including two guiding bars for fingertip and side of finger as shown in Figure 7, which can make the consistent finger area be acquired by our device with reduced misalignment. However, no guiding bar is used in the other device of Figure 8, which is used for collecting the 2nd database. Therefore, we call the 1st and 2nd databases collected by the devices of Figure 7 and Figure 8 as good-quality and mid-quality databases, respectively.

The number of images in the mid-quality database is 1980 (33 people × 2 hands × 3 fingers × 10 trials), and each image has the same size as that of the images in the good-quality database, *i.e.*, 640 × 480 pixels [27].

The last database used in this study is an open finger-vein database (SDUMLA-HMT Finger-vein database) [28], which comprises 3816 images, with a size of 320 × 240 pixels, from 106 people, including six fingers from each person and six trials for each finger. Example images of different trials of one individual (same finger) from each database are given in Figure 9. It can be seen in Figure 9 that the degree of misalignment among the trials of each finger from the mid-quality and open databases is larger than that from the good-quality database.

The accuracies of the finger-vein recognition method were evaluated by performing authentic and imposter matching tests. In our experiments, the images in each finger-vein database could be classified in various ways to allow the discrimination factors to be evaluated. In each experiment, authentic matching tests were used to calculate the pairwise matching distances between the images selected from the same class, whereas for the imposter matching tests, the distances between the pairs of images from different classes were calculated. Assuming that for a particular database, we classify finger-vein images into *M* classes and each class has *N* images, then the number of authentic and imposter matching tests denoted by *A* and *I* are determined by the following Equations (5) and (6), respectively.

(5) A=C2N×M

(6) I=N×C2M×N

where *_N_C*_2_ = *N*(*N* − 1)/2 is the number of two-combinations from a set of *N* elements.

By applying and adjusting the threshold on the matching Hamming distance, we calculated the false acceptance rates (FARs), false rejection rates (FRRs), and the EER. FAR refers to the error rates of imposter matching cases, which are misclassified into authentic classes, whereas FRR refers to the error rates of misclassified authentic testing cases into imposter classes. EER is the error rate when the difference between FAR and FRR is minimized. In addition, we measured the d-prime (*d'*) value, which represents the classifying ability between authentic and imposter matching distributions as the following Equation (7) [3].

(7) $$d\prime =\frac{\mu_{A}-\mu_{I}}{\sqrt{\frac{\sigma_{A}^{2}+\sigma_{I}^{2}}{2}}}$$

where µ_*A*_ and µ_*I*_ represent the mean values of the authentic and imposter matching distributions, respectively, and σ_*A*_ and σ_*I*_ denote the standard deviations of authentic and imposter matching distributions, respectively. A higher d-prime value indicates a larger separation between the authentic and imposter matching distributions, which corresponds to a lower error of recognition, in case that the distributions of authentic and imposter matching scores are similar to Gaussian shape, respectively.

We conducted the following experiments to evaluate the various factors that affect the results of the finger-vein recognition system.

First, we considered each finger of each person to form a different class. This method is used by conventional finger-vein recognition systems to evaluate the recognition accuracy [7,8,9,11,16,17,18,19,20,21,22,23,25]. Consequently, for the good-quality, mid-quality, and open databases, the number of classes were 120 (20 people × 6 fingers), 198 (33 people × 6 fingers), and 636 (106 people × 6 fingers), respectively. As this class definition method includes the dissimilarity information of fingers, hands, and people in the finger-vein database, we considered this as the 1st experiment (classified by fingers, hands, and people).

In the 2nd experiment, we classified the finger-vein images based on people (classified by people), by assuming that the images of all the fingers on both hands from the same person formed the same class. As a result, in the 2nd experiment, the number of classes in each database equaled the number of users, which was 20, 33, and 106 for the good-quality, mid-quality, and open databases, respectively.

In the 3rd experiment, we assumed that the finger-vein images of all the fingers on the left hands of all the people belong to the same class, and those on the right hands of all the people form another class. Thus, there were two classes based on different hand sides in this experiment (classified by hands).

**Figure 10:**
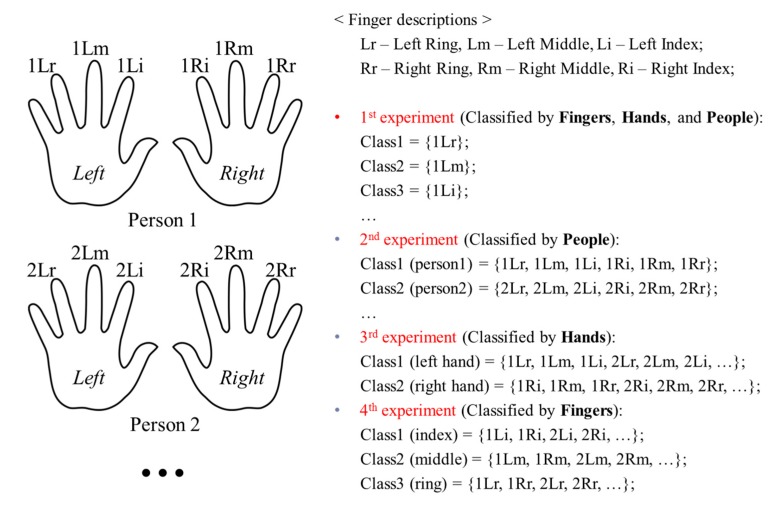
Organization of experiments for finger-vein database.

In the 4th experiment, we evaluated the dissimilarities of finger types by assuming that the images from the index fingers, middle fingers, and ring fingers on both hands of all the people belong to three different classes. This assumption is referred to as (classified by fingers). The organization of these experiments is summarized in Figure 10. The numbers of authentic and imposter matching tests in the experiments on the three databases are determined by Equations (5) and (6), and are shown in Table 2.

**Table 2:** Number of matching tests (authentic and imposter) for the experiments on the three finger-vein databases (*M* is the number of classes in each experiment and *N* is the number of images belonging to one class. Authentic and Imposter refer to the numbers of authentic and imposter matching tests, respectively).

Experiments Databases		1st Experiment	2nd Experiment	3rd Experiment	4th Experiment
		Classified by Fingers, Hands and People	Classified by People	Classified by Hands	Classified by Fingers
Good-quality Database	*N*/*M*	10/120	60/20	600/2	400/3
	Authentic	5400	35,400	359,400	239,400
	Imposter	714,000	684,000	360,000	480,000
Mid-quality Database	*N*/*M*	10/198	60/33	990/2	660/3
	Authentic	8910	58,410	979,110	652,410
	Imposter	1,950,300	1,900,800	980,100	1,306,800
Open Database	*N*/*M*	6/636	36/106	1908/2	1272/3
	Authentic	9540	66,780	3,638,556	2,425,068
	Imposter	7,269,480	7,212,240	3,640,464	4,853,952

**Figure 11:**
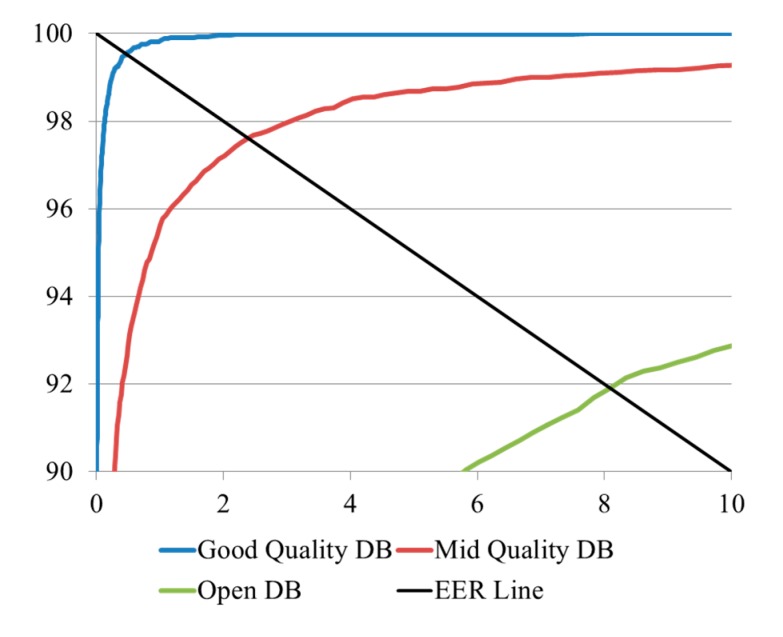
ROC curves of the 1st experiment on the three databases (DBs).

**Figure 12:**
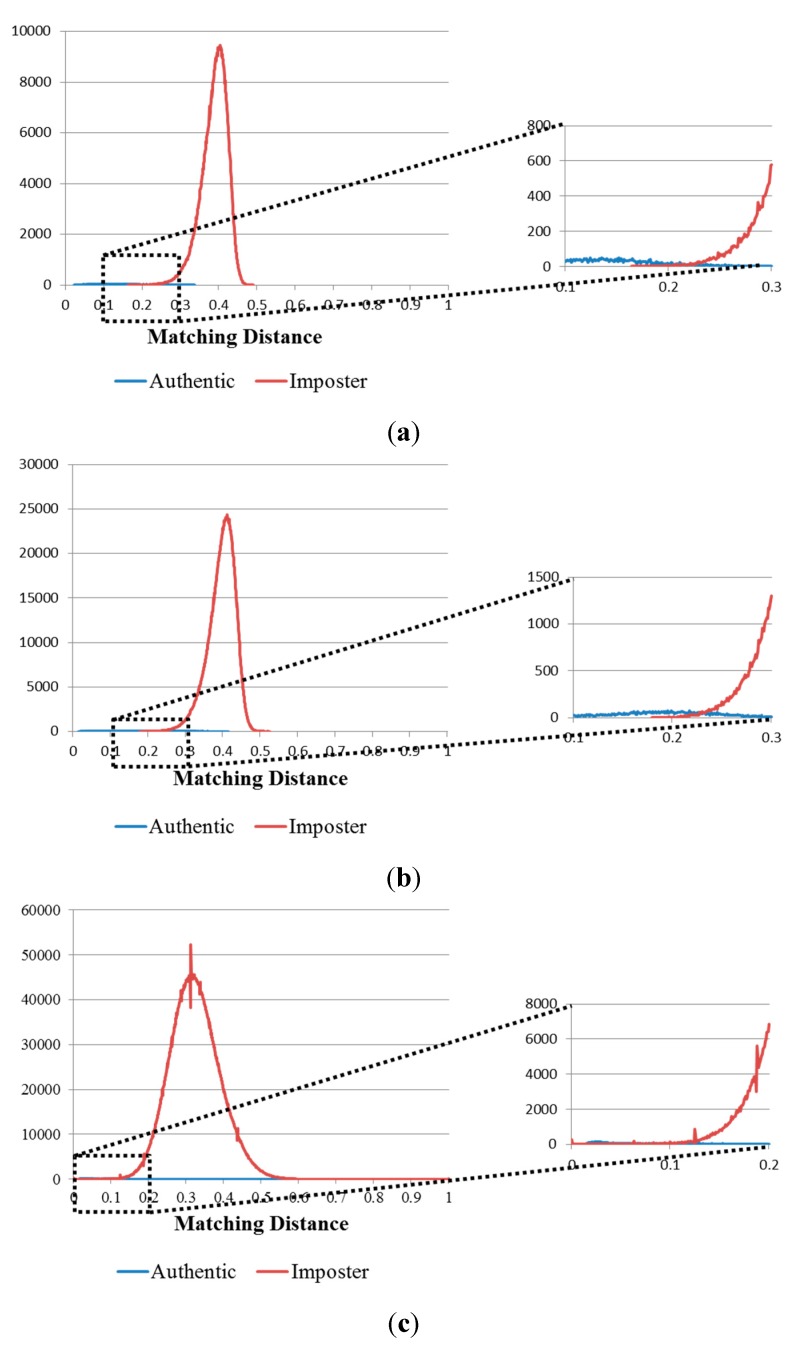
Matching distance distribution of authentic and imposter matching tests in the 1st experiment on the three databases: (**a**) good-quality; (**b**) mid-quality; and (**c**) open database.

Table 3 shows the comparative results of the four experiments defined in Table 2 and Figure 10 for the three databases. In the 1st experiment, in which finger-vein images were classified by fingers, hands, and people, the lowest EER (0.474%) was obtained for the good-quality database. This is due to the fact that this database was captured by the proposed capturing device, which uses a guiding bar to reduce the misalignment among input finger-vein images. In the case of the open database, the authors did not apply any guiding bar for alignment in the image-capturing device [29]. As a result, the EER obtained from this database was the highest (8.096%) because of the misalignment of captured fingers. The results of the first experiment also indicate that the matching accuracies from images in the good-quality database were the highest, followed by those in the mid-quality database, whereas the worst matching accuracies were obtained for the open database, in terms of EERs (0.474%, 2.393%, and 8.096%, respectively). These results correspond to the level of misalignment in each finger-vein database. The resulting plots of the ROC curves and matching distance distributions obtained from the experiments classified by fingers, hands, and people for the three databases are shown in Figure 11 and Figure 12.

**Table 3:** Comparative results of the four experiments for the three databases.

Experiments		Good-Quality Database		Mid-Quality Database		Open Database	
		EER (%)	d-Prime	EER (%)	d-Prime	EER (%)	d-Prime
1st Experiment	Classified by Fingers, Hands, and People	0.474	5.805	2.393	4.022	8.096	2.727
2nd Experiment	Classified by People	40.223	0.695	39.280	0.759	36.095	0.791
3rd Experiment	Classified by Hands	48.427	0.136	49.320	0.072	49.137	0.039
4th Experiment	Classified by Fingers	45.434	0.277	45.506	0.267	47.299	0.147

In the 2nd experiment, we classified the finger-vein images from the three databases based on people. In this way, the finger-vein images from the same person were considered as belonging to the same class; hence, the finger-vein images in different classes indicated the dissimilarities between different people. Likewise, the 3rd and 4th experiments on the three databases, considered images from the same hand side (*i.e.*, either the left or the right hand), and images from the same finger type (*i.e.*, the index, middle, or ring fingers) of all the people to be from the same classes, respectively. A comparison of the results of the three experiments (2nd, 3rd and 4th) on each database by considering the finger-vein dissimilarity between people, hands, and fingers, enabled us to evaluate the effect of each of these factors on the accuracy of the finger-vein recognition system.

**Figure 13:**
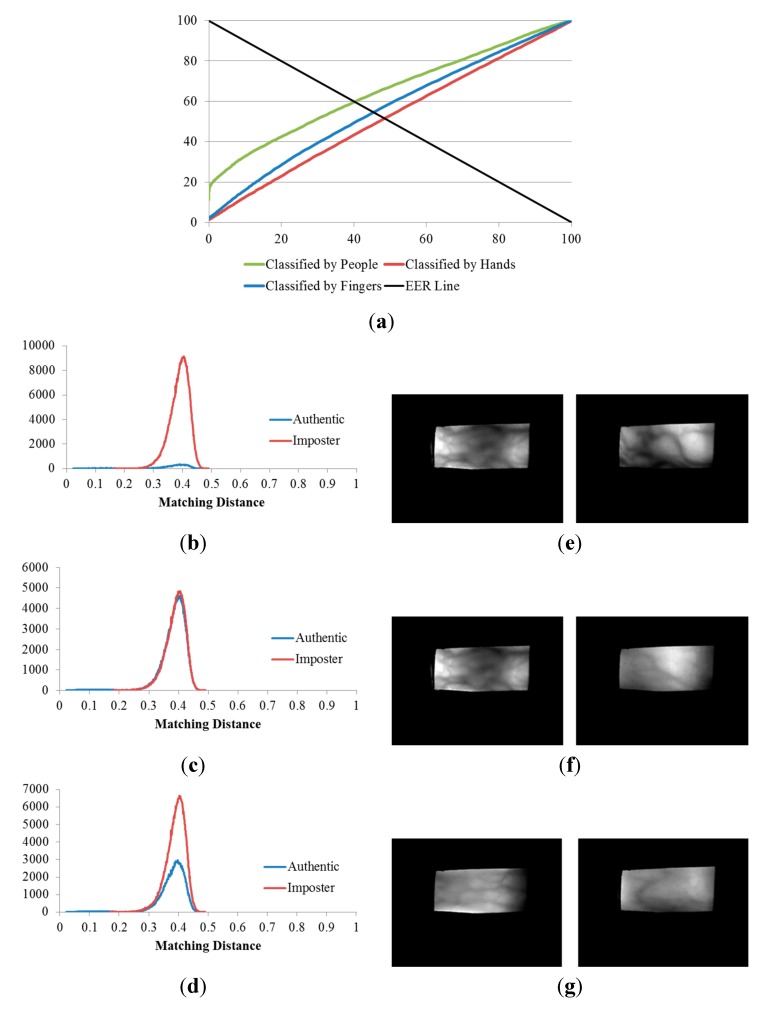
Results of the 2nd, 3rd and 4th experiments on the good-quality database: (**a**) ROC curves of the results of the three experiments; matching distribution of (**b**) the experiment classified by people (2nd experiment); (**c**) the experiment classified by hands (3rd experiment); and (**d**) the experiment classified by fingers (4th experiment), each shown with its corresponding false rejection error case: (**e**) images of the ring and index fingers on left hand of the same person; (**f**) images of the ring and index fingers on left hands of two different people; and (**g**) images of the middle fingers on left and right hands of two different people.

**Figure 14:**
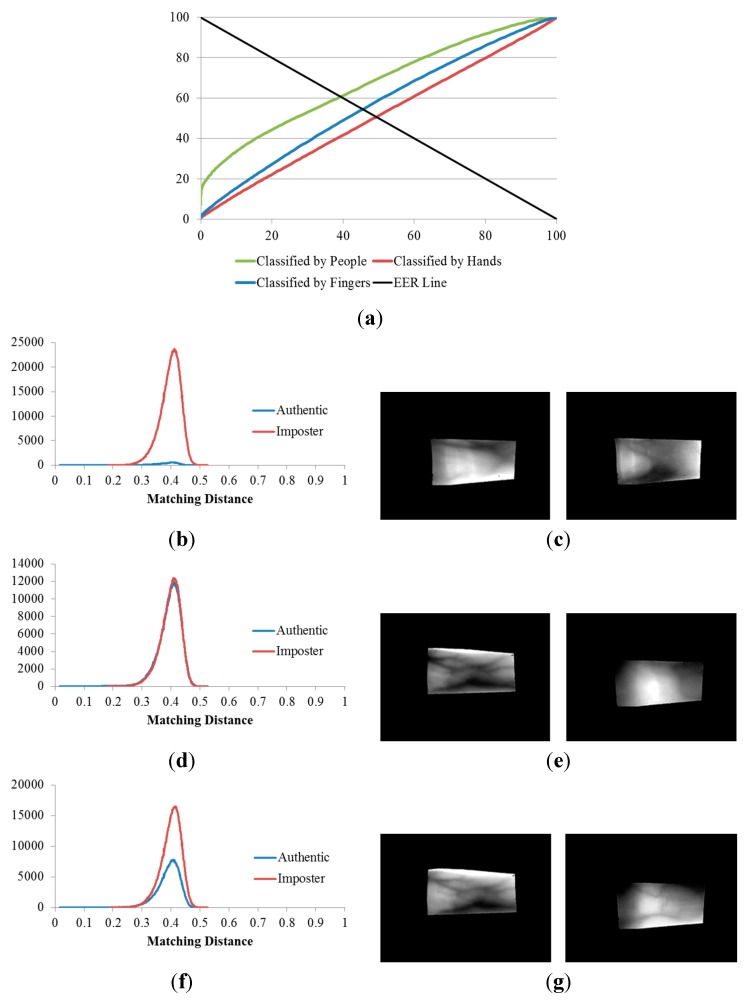
Results of the 2nd, 3rd, and 4th experiments on the mid-quality database: (**a**) ROC curves of the results of the three experiments; matching distribution of (**b**) the experiment classified by people (2nd experiment); (**d**) the experiment classified by hands (3rd experiment); and (f) the experiment classified by fingers (4th experiment), each shown with its corresponding false rejection error case: (**c**) images of the right ring and left middle fingers of the same person; (**e**) images of the ring and index fingers on right hands of two different people; and (**g**) images of the ring fingers on right hands of two different people.

**Figure 15:**
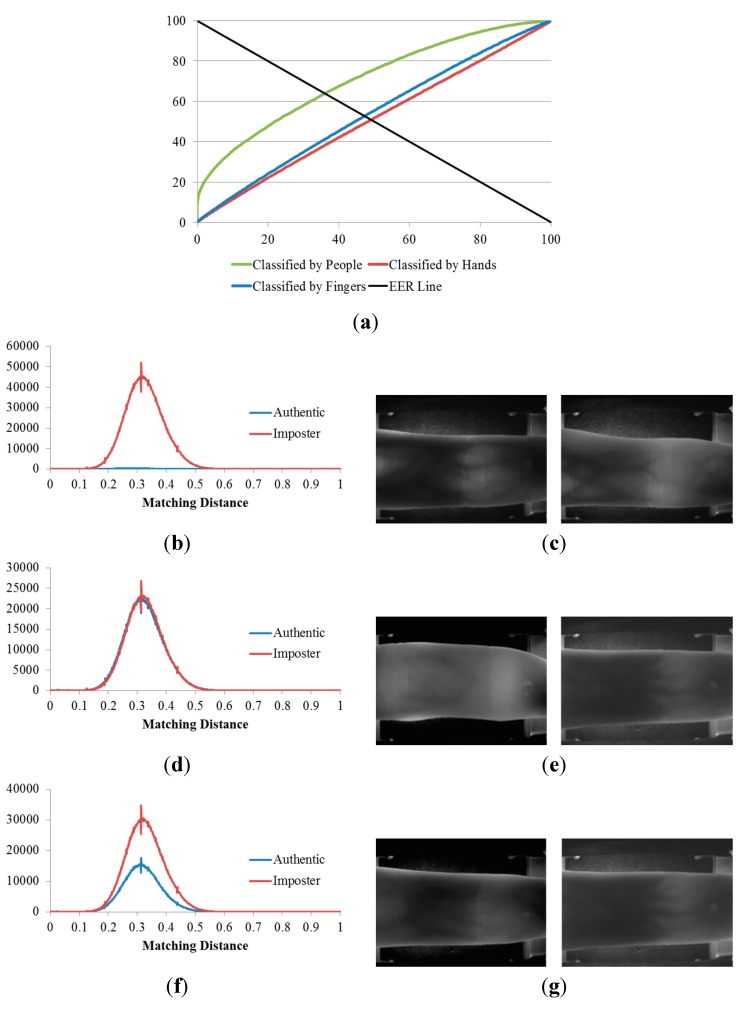
Results of the 2nd, 3rd, and 4th experiments on the open database: (**a**) ROC curves of the results of the three experiments; matching distribution of (**b**) the experiment classified by people (2nd experiment); (**d**) the experiment classified by hands (3rd experiment); and (**f**) the experiment classified by fingers (4th experiment); each shown with its corresponding false rejection error case: (**c**) images of the right middle and right ring fingers of the same person; (**e**) images of the index and middle fingers on left hands of two different people; and (**g**) images of the middle fingers on two hands of two different people.

Table 3 indicates that, when the three databases are compared, the lowest EERs (the highest d-prime value) were obtained from the experiment classified by people (the 2nd experiment), the second lowest EERs (the second highest d-prime value) were obtained from the experiment classified by fingers (the 4th experiment), and that classified by hands (3rd experiment) produced the highest EERs (the lowest d-prime value). This sequence was consistent for all three of the databases.

Consequently, we are able to conclude that, finger-vein dissimilarity increases in the order people, fingers, and hands, respectively. In other words, the discrimination between finger-vein images from different people is larger than that between the different finger types (index, middle, and ring fingers) and that between hands from different sides (left or right hands).

The plots of the ROC curves and matching distributions of authentic and imposter tests obtained from the three experiments (the 2nd, 3rd, and 4th experiments) as well as the error cases for the good-quality, mid-quality, and open databases are shown in Figure 13, Figure 14 and Figure 15, respectively. In the 2nd experiment (classified by people), the cases for which a false rejection was obtained were for different fingers from the same person. The false rejection cases of the 3rd experiment (classified by hands) were the matching pair of vein images of fingers from the same hand side, but belonging to different people or captured from different fingers. Similarly, the false rejections of the 4th experiment (classified by fingers) are cases in which images were captured from the same finger types (*i.e.*, index, middle, or ring fingers) but belonged to different people or hand sides.

### 3.3. Experimental Results Using Multiple Images for Enrollment

**Figure 16:**
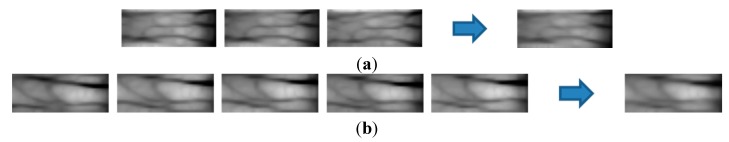
Normalized finger-vein images and their average images when: (**a**) three images; and (**b**) five images were used for enrollment.

In this experiment, we used a number of input finger-vein images for enrollment instead of using only one image as was done previously [7,8,9,11,16,17,18,19,20,21,22,23,25]. The method involving the enrollment of finger-vein data using the average of multiple finger-vein images is as follows. After the input images were captured for enrollment, they were processed and normalized by the methods described in Section 2.2 and Section 2.3. From the image consisting of 50 × 20 pixels, obtained as a result of sub-sampling, we obtained the average image from which we extracted the LBP code which was then enrolled into the system. We applied this method by using either three or five enrollment finger-vein images to compare the matching accuracies with the conventional method, which only uses one image for enrollment. Examples of the average images generated from the 50 × 20 pixel images are shown in Figure 16. The experiments were conducted on the good-quality database as demonstrated in Figure 9a.

**Figure 17:**
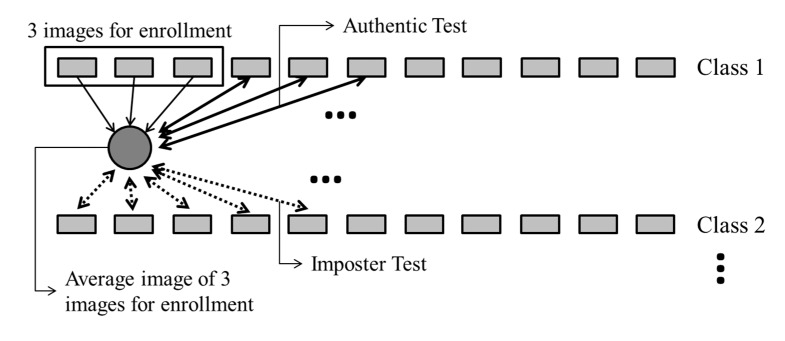
Experimental method when three images were used for finger-vein enrollment.

When three images were used for enrollment, these were selected from the 10 images of each finger of the same user. Then, we extracted the finger-vein code from the average of these three images, and used the data extracted from the remaining seven images of the same finger to perform authentic matching tests. For the imposter matching tests, we used the images of the other fingers to perform matching with the average image generated for the enrolled finger. This experimental method is illustrated in Figure 17.

Assuming that the images from different fingers, hands, and people belong to different classes, the good-quality database contained 120 classes in total, as shown in Table 2. When three images were used for enrollment, the number of authentic matching tests was 100,800 (_10_C_3_ × 7 (the number of remaining images in the same class) × 120 (the number of classes)), whereas the number of imposter matching tests was 17,136,000 (_10_C_3_ × 10 (the number of images in other classes) × 119 (the number of other classes) × 120 (the number of classes from which images for enrollment were selected)).

When five images were used for enrollment, the number of authentic matches was 151,200 (_10_C_5_ × 5 (the number of remaining images in the same class) × 120 (the number of classes)), whereas that of imposter matches was 35,985,600 (_10_C_5_ × 10 (the number of images in other classes) × 119 (the number of other classes) × 120 (the number of classes from which images for enrollment were selected)).

**Figure 18:**
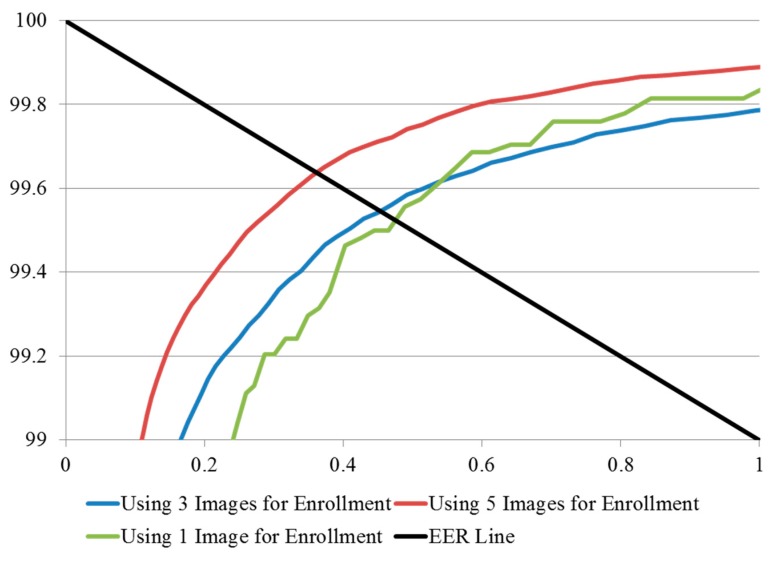
ROC curves of using multiple images for enrollment methods.

**Figure 19:**
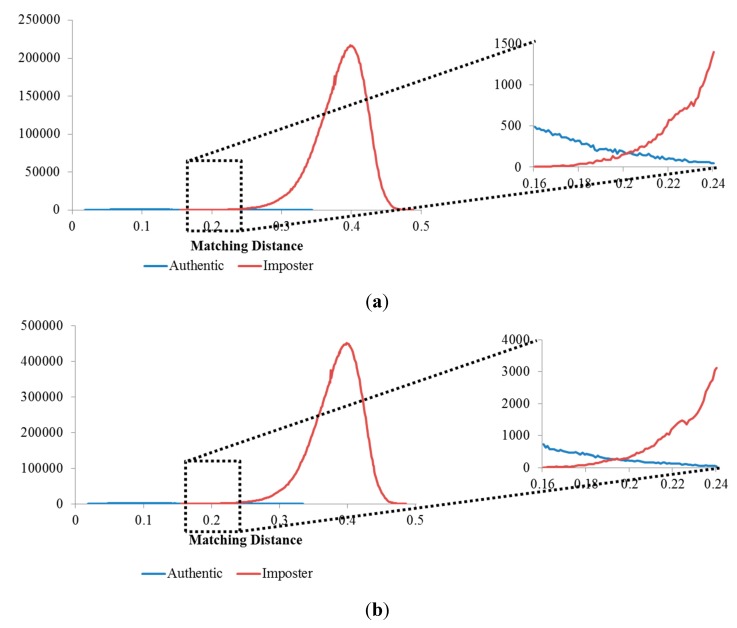
Matching distance distributions of authentic and imposter tests using (**a**) three images; and (**b**) five images for enrollment methods.

The experimental results of the methods in which three and five images were used for enrollment, are compared with those obtained by the conventional method (using one image for enrollment) in Table 4, where it can be seen that the matching accuracy was enhanced by increasing the number of enrollment images, in terms of low EER and high d-prime values. The ROC curves and the distribution plots of authentic and imposter tests corresponding to the results in Table 4 are shown in Figure 18 and Figure 19, respectively, and can be explained as follows. In the finger-vein database, matching errors were mostly caused by misalignment at the time when the input finger-vein images were initially recorded, which subsequently resulted in translation errors in the normalized images of 50 × 20 pixels. The use of image averaging reduced the translation errors within the normalized images and increased the similarities between the enrolled and the matched finger-vein data. Table 5 shows examples of error cases resulting in false rejection when the enrolled images were compared with the test image in the same class, listed according to the number of images used for enrollment.

**Table 4:** Comparative results when multiple images were used for enrollment.

Number of Images for Enrollment	EER (%)	d-Prime
1	0.474	5.805
3	0.454	6.406
5	0.362	6.633

**Table 5:** False rejection cases: Comparison of the detected input finger-vein images with the enrolled images.

Number of Images for Enrollment	Enrolled Images	Average Image for Enrollment	Input Image
1	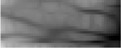	N/A	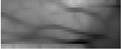
	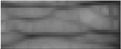	N/A	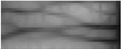
3	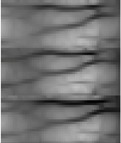	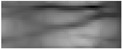	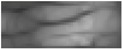
5	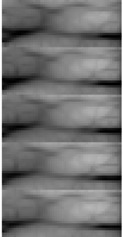	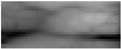	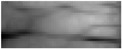

From Table 5, we can see that, when one image was used for finger-vein enrollment, false rejection was mostly caused by translational errors between images of the same finger. These errors can either occur during translations in the horizontal direction of the image (the first row of Table 5) or in the vertical direction of the image (the second row of Table 5).

The reason why false rejections occur when either three or five images are used for enrollment is as follows. The misalignment between finger-vein images selected for enrollment resulted in blurred vein lines and the appearance of artifacts in the average images that were generated. Consequently, this led to high matching distances between the enrolled finger-vein data and test data, and these cases were misclassified into the imposter matching class.

### 3.4. Experimental Results Using Score-level Fusion Methods with Multiple Input Images

In the final set of experiments, we evaluated the matching accuracies and classifying ability of the system by using score-level fusion methods with multiple input images. This involved the application of SUM and PRODUCT rules, of which the formulas are expressed by Equations (8) and (9), to combine either three or five matching scores, which were then used to classify images as being either authentic or those of an imposter.

(8) $$\begin{array}{ccc} SUM\;rule: & & d_{S}=\sum_{i=1}^{N}d_{i} \end{array}$$

(9) $$\begin{array}{ccc} PRODUCT\;rule: & & d_{P}=\prod_{i=1}^{N}d_{i} \end{array}$$

where *d_i_* is the original matching score between the *i*th input finger-vein image and the one that was enrolled, and *d_S_* and *d_P_* are the resulting matching scores obtained by using the SUM and PRODUCT rules, respectively. *N* (3 or 5) is the number of scores to be combined.

The experiments were conducted on the good-quality database of Figure 9a as follows. From the 10 finger-vein images of each finger of an individual person in the database, we selected either three or five images as the authentic test images and used the remaining seven or five images as the enrolled images, respectively. For the imposter tests, we considered each of the 10 images of the other fingers in the database as the enrolled finger-vein image. For each enrolled image, we calculated the matching scores with the test images, combined these scores using the SUM and PRODUCT rules, and used the fused scores for final decisions. The use of this experimental method produced the same number of authentic and imposter-matching test results as for the experiments in which multiple images were used for enrollment in Section 3.3. That is because the number of images used for enrollment in the previous experiments (Section 3.3) and the number of scores used for score-level fusion in these experiments were the same, *i.e.*, three and five. Therefore, for each of the rules, SUM and PRODUCT, when three scores were used for fusion purposes, the numbers of authentic and imposter tests were 100,800 and 17,136,000, respectively, whereas the use of the five-score-level fusion method produced 151,200 authentic matches and 35,985,600 imposter matches.

**Figure 20:**
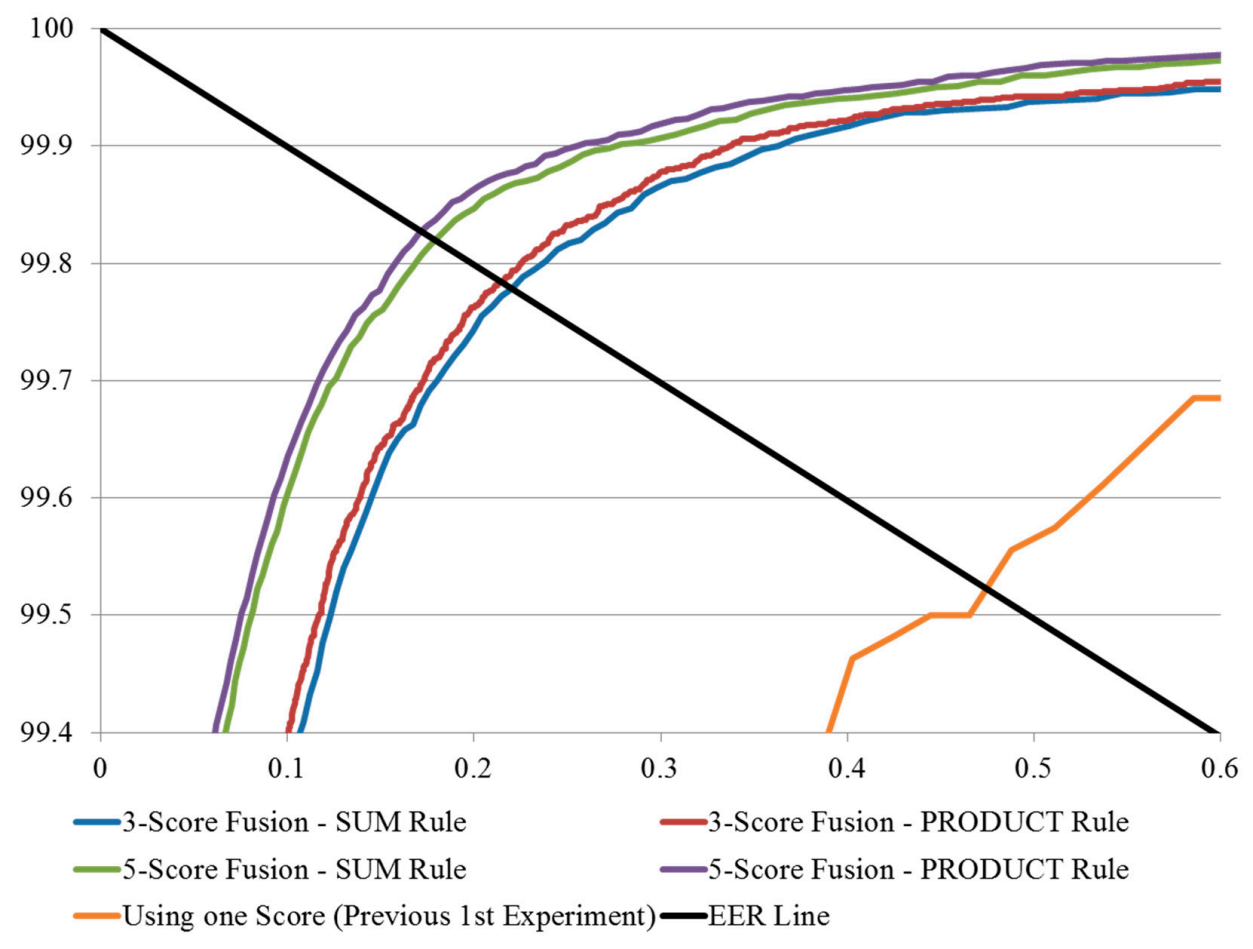
ROC curves obtained from the experiments with or without score-level fusion (the 1st experiment of Table 3) on good-quality database.

**Figure 21:**
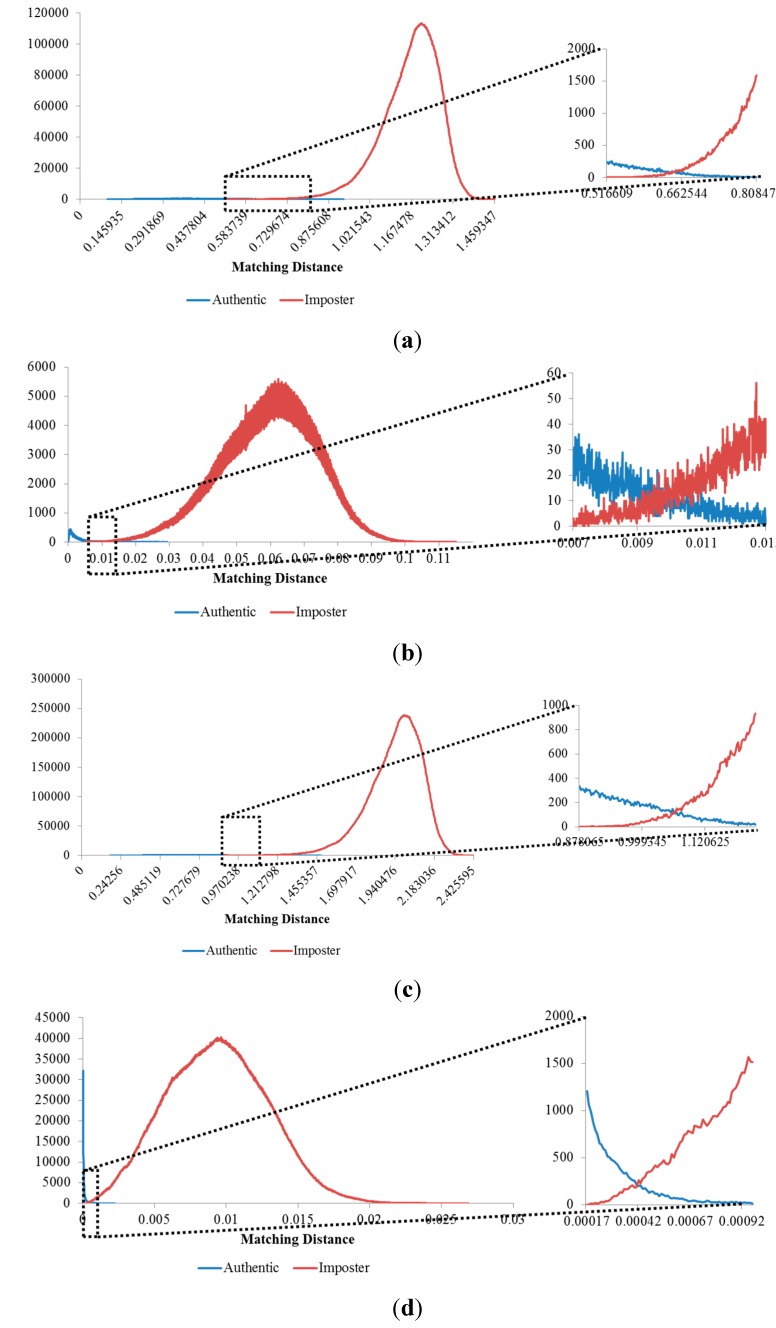
Matching distance distributions of authentic and imposter tests using score-level fusion methods: Three-score fusion method (**a**) using the SUM rule; and (**b**) using the PRODUCT rule; five-score fusion method (**c**) using the SUM rule; and (**d**) using the PRODUCT rule.

The experimental results of the score-level fusion methods were compared with the previous experiment in which one score was used for finger-vein recognition (the 1st experiment performed on the good-quality database of Table 3 in Section 3.2) and shown in Table 6. The ROC curves and the distribution plots of the authentic and imposter matching scores obtained from these experiments are shown in Figure 20 and Figure 21.

**Table 6:** Comparisons of the matching accuracies of the score-level fusion methods and the previous matching method in which one finger-vein image was used.

Number of Score	Score-Level Fusion Rule	EER (%)	d-Prime
1	N/A	0.474	5.805
3	SUM	0.220	6.892
	PRODUCT	0.215	5.573
5	SUM	0.180	7.183
	PRODUCT	0.172	3.755

It can be seen from Table 6 that the score-level fusion methods enhanced the matching accuracies in that they resulted in low EER values and that the best results (the EER of 0.172%) were obtained in case that five matching scores were fused with the PRODUCT rule. In the case of using the same number of fused scores, the PRODUCT rule produced a lower EER value compared to the SUM rule. However the d-prime value of PRODUCT rule was lower than that of SUM rule. In general, the case of lower EER produces that of the higher d-prime value only in the case that the authentic and imposter distributions are similar to Gaussian shape, respectively. However, the authentic distributions obtained by PRODUCT rule of Figure 21b,d are different from the Gaussian shape, which causes the d-prime value not to correctly reflect the accuracy of finger-vein recognition. Therefore, the d-prime value of PRODUCT rule was inconsistently lower than that of SUM rule in Table 6.

### 3.5. Discussions

Regarding the issue of using average images for feature extraction as shown in Figure 16 and Figure 17, the method of selecting one enrolled image (whose finger-vein code shows the minimum distances compared to the codes of other enrolled images) has been most widely used (1st method). However, the finger-vein code of one image among three or five enrolled images for enrollment is selected by this method, which cannot fully compensate for the differences among three or five enrolled images. Therefore, we adopt the method of using average image for enrollment as shown in Figure 16 and Figure 17 (2nd method). To prove this, we compared the accuracy of finger-vein recognition by this 1st method with that by the 2nd method. The EER (d-prime) by the 1st method with three and five images for enrollment are 0.468% (6.128) and 0.412% (6.597), respectively. By comparing the EER (d-prime) by the 2nd method as shown in the 3rd and 4th rows of Table 4, we confirm that our 2nd method using average image for enrollment outperforms the 1st method.

The method of simply averaging the images for enrollment can be sensitive to image alignment and detailed features can be lost in the average image. In order to solve this problem, in our research, the misalignment among the images was firstly compensated by template matching before obtaining the average image. For example in Table 5, in the case that the number of images for enrollment is 3, the horizontal and vertical movements of the second enrolled image based on the first one are measured by template matching with the first enrolled image. If the measured horizontal and vertical movements of the second enrolled image are −2 and −1 pixels, respectively, for example, the compensated image is obtained by moving the original second enrolled image by +2 and +1 pixels, respectively, in the horizontal and vertical directions. From this, we can obtain the (compensated) second enrolled image where the misalignment based on the first enrolled image is minimized. Same procedure is iterated with the third enrolled image. From this procedure, we can obtain three (compensated) enrolled images where the misalignment between each other is minimized, and these three images are averaged for obtaining one enrolled image. Therefore, we can solve the problem that the average image is sensitive to image alignment and detailed features can be lost in the average image.

The total number of images in the good-quality database was 1200 (20 people × 2 hands × 3 fingers × 10 images), and that in the mid-quality database is 1980 (33 people × 2 hands × 3 fingers × 10 images). In order to obtain the meaningful conclusions and prove our conclusion irrespective of kinds of database, we also include the third open database for experiments. The total number of images in the open database was 3816 (106 people × 2 hands × 3 fingers × 6 images). Consequently, a total of 6996 images were used for our experiments, and we obtained the conclusion through a great deal of authentic and imposter matching, as shown in Table 2.

**Figure 22:**
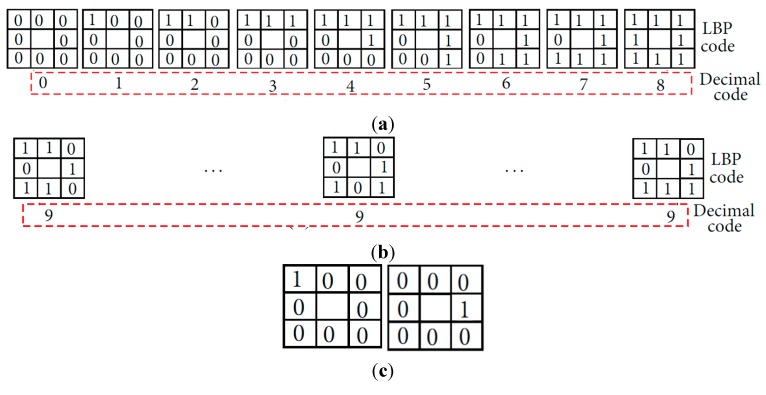
Example of uniform and nonuniform patterns and their assigned decimal codes by uniform LBP, respectively: (**a**) uniform patterns; (**b**) nonuniform patterns; (**c**) two cases of decimal code of 1 by uniform LBP.

The original LBP used in our method can be more sensitive to noise than the uniform LBP. Therefore, in our method, the sub-sampled image of 50 × 20 pixel is used for feature extraction by LBP as shown in Figure 4c,f,i, which can reduce the noise in the image for feature extraction. In addition, the two cases of LBP codes in Figure 22c are assigned as same decimal code of 1 by the uniform LBP although they are actually different LBP code (00000001 (left case) and 00010000 (right case)), which can reduce the dissimilarity between two different patterns of finger-vein image. Therefore, we use the original LBP method in our research.

We compared the accuracies by our original LBP and those by uniform LBP. The EER (d-prime) by uniform LBP with 1st, 2nd, and 3rd databases are 0.925% (5.122), 4.513% (3.443), and 12.489% (2.114). The EERs by uniform LBP are larger than those by original LBP of the 1st row of Table 3. In addition, the d-prime values by uniform LBP are smaller than those by original LBP of the 1st row of Table 3. From that, we can confirm that the performance by our original LBP is better than that by uniform LBP.

## 4. Conclusions

This paper proposed a new finger-vein capturing device that relies on accurate finger positioning to reduce misalignment when vein images are captured. This device was used to capture images to construct a database composed of good-quality finger-vein images, which were compared to the images in the mid-quality database (which was used in previous research) and an open database. The images in the good-quality database produced lower matching EER and higher d-prime values. Based on the comparative experimental results considering finger-vein dissimilarities between people, hands, and fingers in the three databases, we evaluated the factors that affect the accuracy of finger-vein recognition and concluded that finger-vein dissimilarity decreases for people, fingers, and hands in that order. We also proposed a method based on the use of multiple images to generate an image for finger-vein enrollment, instead of using one image as done previously. For our final set of experiments, we proposed a recognition method using score-level fusion obtained by using SUM and PRODUCT rules. The experimental results obtained for images from the database captured by our device, showed that the use of multiple enrollment images and score-level fusion could enhance the matching accuracies by reducing the EER. For future work, we plan to evaluate the various factors determining the accuracies of hand vein or palm vein recognition systems. In addition, we would also consider evaluating the effect of race, age, and gender on the accuracy of vein recognition.
